# Disappearance of myocardial perfusion defects on prone SPECT imaging: Comparison with cardiac magnetic resonance imaging in patients without established coronary artery disease

**DOI:** 10.1186/1471-2342-9-16

**Published:** 2009-08-10

**Authors:** Bo Hedén, Eva Persson, Marcus Carlsson, Olle Pahlm, Håkan Arheden

**Affiliations:** 1 Department of Clinical Physiology, Lund University Hospital, Lund, Sweden

## Abstract

**Background:**

It is of great clinical importance to exclude myocardial infarction in patients with suspected coronary artery disease who do not have stress-induced ischemia. The diagnostic use of myocardial perfusion single-photon emission computed tomography (SPECT) in this situation is sometimes complicated by attenuation artifacts that mimic myocardial infarction. Imaging in the prone position has been suggested as a method to overcome this problem.

**Methods:**

In this study, 52 patients without known prior infarction and no stress-induced ischemia on SPECT imaging were examined in both supine and prone position. The results were compared with cardiac magnetic resonance imaging (CMR) with delayed-enhancement technique to confirm or exclude myocardial infarction.

**Results:**

There were 63 defects in supine-position images, 37 of which disappeared in the prone position. None of the 37 defects were associated with myocardial infarction by CMR, indicating that all of them represented attenuation artifacts. Of the remaining 26 defects that did not disappear on prone imaging, myocardial infarction was confirmed by CMR in 2; the remaining 24 had no sign of ischemic infarction but 2 had other kinds of myocardial injuries. In 3 patients, SPECT failed to detect small scars identified by CMR.

**Conclusion:**

Perfusion defects in the supine position that disappeared in the prone position were caused by attenuation, not myocardial infarction. Hence, imaging in the prone position can help to rule out ischemic heart disease for some patients admitted for SPECT with suspected but not documented ischemic heart disease. This would indicate a better prognosis and prevent unnecessary further investigations and treatment.

## Background

Myocardial perfusion single-photon emission computed tomography (SPECT) is widely used to diagnose or exclude coronary artery disease. A normal perfusion SPECT result is associated with excellent prognosis [1], and the sensitivity and specificity are high for detection of stress-induced ischemia [2]. Imaging is traditionally performed in supine position, which may cause attenuation artifacts, mostly located inferiorly in men and anteriorly in women [3]. The attenuation artifacts may simulate perfusion defects, leading to false-positive identification of myocardial infarction [4].

Since it is of great importance to exclude infarction in patients without previously identified coronary artery disease who do not have stress-induced ischemia, different techniques such as gated SPECT and attenuation correction are used to differentiate artifacts from infarction [5,6]. In addition, imaging in the prone position has been suggested as a method to prevent misleading interpretation of myocardial perfusion SPECT imaging [4,7-9]. Compared to the supine position, acquisition in the prone position generates higher counts from the inferior wall [8,10,11]. Clinical studies have shown that prone imaging has significantly higher specificity and accuracy for right coronary artery disease compared to supine imaging [11,12]. Patients with inferior-wall defects on supine images that are absent on prone images have been shown to have a low risk of cardiac events, similar to that of patients with normal supine-only studies [13]. Therefore, a defect present in the supine position that disappears in the prone position may well be assumed to reflect attenuation and not myocardial infarction [3]. However, there are no studies specifically showing the absence of myocardial infarction in patients with "disappearing" defects in prone imaging. Delayed contrast enhanced cardiac magnetic resonance (CMR) imaging has emerged as the gold standard for infarct detection in recent years [14,15]. The ability of CMR to detect, localize and quantify fibrosis and infarction offers the opportunity to examine if prone SPECT imaging can be used to exclude myocardial infarction.

Therefore, the aim of this study was to investigate to what extent fixed defects in the supine position that disappear in prone position, and fixed defects that persist in the same location on both supine and prone images, represent attenuation or myocardial infarction in patients without known coronary artery disease and no stress-induced ischemia. Cardiac magnetic resonance (CMR) imaging with delayed contrast enhancement was used as the "gold standard" for identifying myocardial infarction and fibrosis.

## Methods

### Study population

Patients referred to Lund University Hospital for elective myocardial perfusion SPECT because of suspected coronary artery disease, i.e. due to intermittent chest discomfort were considered for the study. Patients were included if they had a defect on the SPECT stress image in the supine position that could represent myocardial infarction but no history of myocardial infarction on ECG or in the patient history. Patients with stress-induced ischemia were excluded because they revealed evidence of coronary artery disease, as were patients with left bundle branch block, cardiac pacemaker, severe arrhythmia, or claustrophobia. Prone images were acquired post stress only, which in principle yields the same result concerning fixed defects as resting images, since patients with stress-induced ischemia were excluded. No patients were excluded due to problems with lying in the prone position. The local medical ethics committee approved the study. All patients gave written informed consent, and 52 patients were included in the study.

### SPECT imaging protocols

A 2-day protocol was used. On day 1, after physiological or pharmacological stress, supine acquisition was obtained, immediately followed by prone acquisition. Within 4 days, supine acquisition was repeated at rest according to the routine clinical procedure. Patients were injected intravenously with a body-weight-adjusted dose (450 to 820 MBq) of ^99m^Tc-tetrofosmin during stress. Physiological stress was induced using a bicycle ergometer. Patients exercised to peak ability and to at least 85% of the maximum predicted heart rate. ^99m^Tc-tetrofosmin was injected 1 minute before peak exercise. Pharmacological stress was performed with adenosine infusion (140 μg/kg/min) for 5 minutes, ^99m^Tc-tetrofosmin was injected after 3 minutes of infusion. Exercise on bicycle during adenosine infusion was performed when possible, to diminish the side effects of adenosine. A 12-lead ECG was acquired during the whole stress procedure.

Acquisition was performed about 45 to 60 minutes after tracer injection, using a dual-head gamma camera (Vertex, ADAC Corporation, Milpitas, CA, USA) equipped with high-resolution, parallel-hole collimators. Data were collected at 32 projections over a 180° orbit, 40 seconds per projection, and 64 × 64 matrix zoomed to a pixel size of 5 mm. ECG-gated SPECT acquisition in eight phases (bins) was performed on 50 patients. In 2 patients gated SPECT could not be acquired because of atrial fibrillation. The acquisition protocol has previously been described in detail [16]. Attenuation correction was not used. SPECT images were reconstructed and post-filtered (Butterworth order, 5.0; cut-off frequency, 0.6 for supine images and 0.66 for prone images. The supine images were gated and the prone images un-gated). The SPECT reconstruction and reorientation were automated (AutoSPECT+™ using Instill motion correction, ADAC, Milpitas, CA, USA), but checked by an experienced operator who made corrections when needed.

### Acquisition and analysis of CMR

At a mean of 31 ± 29 days after the stress supine/prone SPECT imaging, the patients underwent CMR in the supine position using a 1.5 T system (Philips Intera CV, Philips, Best, The Netherlands) with a cardiac synergy coil. Short- and long-axis delayed contrast-enhanced CMR images covering the left ventricle were acquired 15 to 20 minutes after administration of 0.2 mmol/kg of an extra-cellular gadolinium-based contrast agent (Magnevist^®^, Gd-DTPA, Schering Nordiska AB, Järfälla, Sweden). Acquisition was performed during breath-hold, using an ECG-triggered segmented inversion-recovery gradient echo sequence. Typical imaging parameters were: repetition time 4.2 ms, echo time 1.3 ms, flip angle 15°, SENSE factor 2, slice thickness 8 mm. Myocardial infarction/scarring were detected as hyper-enhanced regions in the left ventricle visualized in two perpendicular imaging planes [14,15]. Cine images in the short-axes plane and three long-axis planes; the two-chamber, four-chamber and left ventricular outflow tract views were obtained in all subjects during end-expiratory apnea using a steady state free precession sequence. Typical imaging parameters were: repetition time 2.9 ms, echo time 1.5 ms, flip angle 60°, SENSE factor 2, slice thickness 8 mm. Cine images were used to determine regional and global function.

### Image analysis

Two physicians evaluated the supine and prone SPECT images in consensus and blinded to patient data and CMR results. The criteria used for describing defect location on the supine images was the model proposed by Cerquiera et al [17]. However, for clarity the results are presented as the anterior, lateral, inferior and septal wall as well as apex, where the anterior wall represents segment 1, 7 and 13 according to Cerquiera; the lateral wall 5, 6, 11, 12 and 16; the inferior wall 4, 10 and 15; the septal wall 2, 3, 8, 9 and 14; and the apex segment 17. Thereafter, the prone images were evaluated and the defects on the supine images were classified as remaining or disappearing. The improvement from supine to prone had to be complete to be considered as disappearing. If the defect seen in supine imaging disappeared but a new defect was seen the defect was classified as disappearing; a biological perfusion defect is not likely to change localization from supine to prone imaging. One additional experienced physician, blinded to patient data and CMR results, also evaluated all supine and prone SPECT images. Assessment differed in 3 cases, and these were resolved by adjudication in conference. The gated SPECT images were evaluated on the supine SPECT images in consensus by two physicians. If a perfusion defect had normal regional function it was classified as artifact and if the regional function was decreased it was classified as infarct. Two experienced physicians, blinded to patient data and SPECT results, evaluated the CMR images visually in consensus. The regions of myocardial fibrosis/infarction and regional dysfunction were determined as anterior, lateral, inferior, septal or apical. Myocardial fibrosis/infarction was quantified using Segment v1.6 http://segment.heiberg.se/[18].

### Statistical analysis

Continuous data are expressed as mean ± SD. The results from SPECT and CMR imaging are presented in a 2 × 2 frequency table. Negative and positive predictive values were calculated. The 95%-confidence interval (CI) was estimated with the assumption of binomial distribution. The concordance between the SPECT and CMR imaging were calculated for table 1 and 2 respectively.

**Table 1:** Defects on Supine Imaging: Correlation with Presence on Prone Imaging and CMR in the Same Region

n = 63 supine defects	No Fibrosis/infarction by CMR	Fibrosis/infarction by CMR
Disappearing Defect by Prone SPECT	37	0
Remaining Defect by Prone SPECT	22	4

SPECT = myocardial perfusion Single Photon Emission Computed Tomography.

CMR = Cardiac Magnetic Resonance imaging.

**Table 2:** Defects on CMR Imaging: Correlation with number of patients with abnormal function on SPECT gated acquisition.

n = 50 gated patients	No Fibrosis/infarction by CMR	Fibrosis/infarction by CMR
Normal function by gated SPECT	36	3
Abnormal function by gated SPECT	7	4

SPECT = myocardial perfusion Single Photon Emission Computed Tomography.

CMR = Cardiac Magnetic Resonance imaging.

## Results

The characteristics of the included 52 patients are listed in Table 3. There were 29 females, 59% of the study population. None of the patients had a cardiac event between the myocardial perfusion SPECT imaging and the CMR study according to patient records.

**Table 3:** Characteristics of the Study Population

Characteristics	Data (n = 52)
Age (y)	59 ± 10
Female sex	29 (56%)
Body-mass index (kg/m^2^)	27.4 ± 3.9
Hypertension*	15 (29%)
Medications	
Beta-blockers	20 (38%)
Angiotensin-converting enzyme inhibitors	12 (23%)
Calcium blockers	12 (23%)
Statins	14 (27%)
Diuretics	12 (23%)
Anticoagulants	22 (42%)

Data are presented as mean ± SD or number (%).

* = According to medical records.

**Figure 1:**
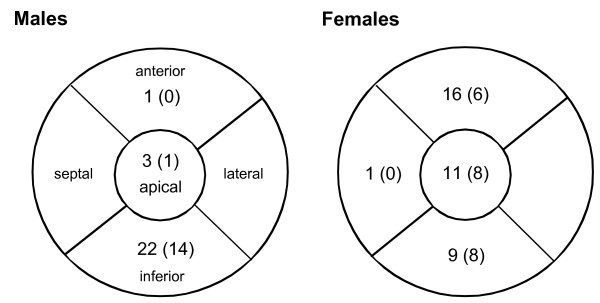
**The number of defects in initial supine imaging by myocardial regions for male (left) and female patients (right)**. The numbers of defects that disappeared in prone imaging are in parentheses. Of the 26 defects seen in the male population (left circle), 22 (85%) were seen in the inferior wall, 3 (12%) were apical defects and only 1 was in the anterior region of the left ventricle. Of the 22 inferior defects, 14 (64%) disappeared in the prone position and of the 3 apical defects 1 disappeared. The anterior defect persisted in prone position. Of the 37 defects seen in the female population (right circle) 16 (43%) were seen in the anterior wall, 11 (30%) were located apically and 9 (24%) were inferior defects. In the female population most defects disappeared in the inferior wall, 8 out of 9 (89%), 8 of the 11 apical defects also disappeared (73%) but only 6 of the 16 anterior defects disappeared (38%). The one septal defect persisted.

Sixty-three areas with defects were seen in 52 patients. Of these defects, 37 (59%) were seen in the female population and 26 (41%) in the male population. The number of defects thus reflects the percentage of included females vs males. Anterior/apical defects predominated in women, 27 of the 37 defects (73%) were seen in the anterior/apical region. In men, 22 of the 26 defects (85%) were located in the inferior left ventricular wall (Figure 1).

**Figure 2:**
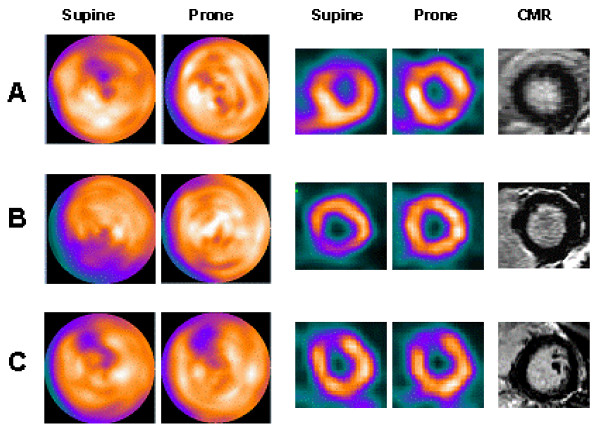
**Comparison of supine and prone SPECT imaging with corresponding CMR imaging**. The 2 left columns show SPECT bull's-eye plots; the 2 middle columns representative SPECT short-axis images; and the right column the corresponding CMR images. The prone polar maps represent raw data and are not normalized to a normal database. **A**: a female patient with an apparent anterior-wall defect in the supine position that disappears in the prone position, CMR was normal (no signs of infarction). **B**: a male patient with an apparent inferior-wall defect in the supine position that disappears in the prone position, corresponding with a normal CMR. **C**: a defect that persists from supine to prone position in a female patient. This patient had normal CMR results. A and B are examples of the prone images correctly excluding myocardial infarction, whereas C is an example of a prone image that failed to correct for an attenuation artifact.

**Figure 3:**
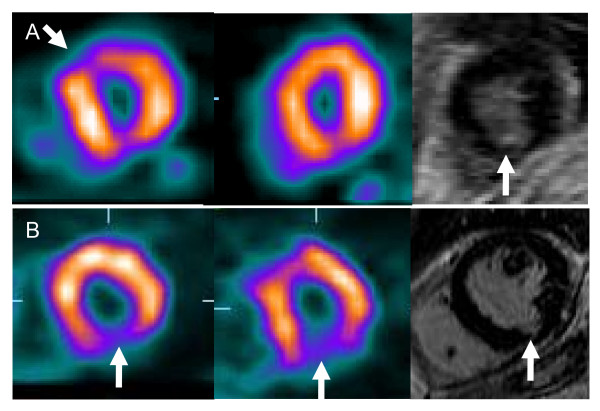
**Two patients with myocardial infarction on CMR**. **A**. A 74-year old woman with an inferior subendocardial infarction. The minor inferior perfusion defect was not read as a true defect, but the patient was included because of the anterior defect in supine imaging. The infarct did not cause decreased function on gated SPECT. **B**. A 69-year old man with an inferior subendocardial infarction detected on prone and supine SPECT as a perfusion defect.

Of the 63 defects, 37 disappeared in prone imaging, 58% in the male population and 59% in the female population (Table 1). In these cases, the prone image correctly excluded myocardial fibrosis/infarction, since no patient had fibrosis/infarction by CMR in the corresponding region (Figure 2). However, in one patient the SPECT was read as normal in the inferior region, both in supine and prone position but showed a small inferior subendocardial infarction (estimated weight: 3 g) (Figure 3). Thus the negative predictive value for myocardial infarction was 97% (CI 84%–100%).

When assessing the data with regard to location of the defect, 22 (71%) of the 31 inferior defects disappeared on prone imaging, as did 9 (64%) of the 14 apical defects. For the 17 anterior defects, however, only 6 (35%) disappeared in prone position. These defects were mostly seen in females.

There were 26 defects that remained in the same location in prone position (Table 1). In only one of these, CMR confirmed myocardial infarction with ischemic pattern in the corresponding inferior region. In addition, three patients showed abnormalities on the CMR; one patient demonstrated infarction in an adjacent region, one patient showed inferoseptal and inferolateral epicardial fibrosis as in myocarditis, and one patient showed signs of non-compaction cardiomyopathy with apical thinning, dysfunction and an abundance of trabeculation. The positive predictive value of a remaining defect for myocardial infarction was therefore 8% (CI 1%–25%). The positive predictive value for a remaining defect being caused by any myocardial fibrosis/infarction was 15% (CI 2%–34%). The other 22 defects were thereby considered artifacts, even though there were defects in both supine and prone images, since CMR showed no sign of myocardial fibrosis/infarction and there was normal function in that area (Figure 2). Therefore, despite the very high value of a disappearing defect by prone SPECT to predict no injury, the kappa value for the concordance between SPECT and CMR is only 0.18 (CI -0.10 – 0.45), with 65% correctly classified defects (CI 52 – 77%). The concordance between SPECT and CMR concerning fibrosis/infarction and regional function was 0.33 (CI -0.04 – 0.70) with 80% correctly classified patients (CI 66 – 90%).

CMR also identified fibrosis at the site of the right ventricular insertion to the septum in two patients which can be seen in pulmonary hypertension and dilated cardiomyopathy. These patients had normal perfusion on SPECT imaging, as did one patient that was read normal in both supine and prone imaging, but showed a small inferior infarction on CMR.

When gated SPECT were used to differentiate between myocardial infarction and artifact, 4 of the 7 patients with fibrosis/infarction detected with MR had either regional or global dysfunction in the SPECT images, thus SPECT correctly indicated myocardial fibrosis/infarction. However the remaining 3 patients with fibrosis/infarction on CMR had no regional wall dysfunction on gated SPECT, thus 3/7 (43%) of the fibrosis/infarction would be missed. Furthermore 7 of the other 45 patients who showed no scars on CMR, had global or regional wall motion abnormalities in the SPECT images (Table 2).

There were no visual differences in defect size in the patients reconstructed with and without motion correction.

## Discussion

This study has demonstrated that defects on supine SPECT imaging that disappeared on prone imaging were not caused by myocardial infarction. However, defects that remained from supine to prone SPECT imaging reflected myocardial fibrosis/infarction in only 15%, in this population of patients with no history of myocardial infarction. There was no gender difference in the usefulness of prone imaging, even though the location of the defects differed between males and females.

There is an ongoing debate on whether attenuation correction, gated SPECT or prone imaging is the best technique to avoid artifacts in clinical patients [5,6]. Prone imaging does not require any additional hardware but on the other hand an additional scan. Gated SPECT is acquired during the perfusion scan and can be used in conjunction with prone imaging and attenuation correction in most patients. Attenuation correction requires specialized hardware and may prolong the acquisition. Of the three, attenuation correction is the most expensive technique.

This study has shown the previously unproven notion that defects in the supine position that disappear in the prone position represent attenuation artifacts. This is valuable knowledge in the clinical setting, since it is of great importance to exclude myocardial infarction in patients without previously identified coronary artery disease and who do not have stress-induced ischemia.

A study by Hayes and colleagues showed a similar, low risk of cardiac events among patients with inferior defects on supine imaging that disappeared on prone imaging compared with those who had normal supine only studies [13].

Previous studies have explained the disappearance of artifacts in the prone position. Acquisition with the patient in the prone position generates higher counts from the inferior wall than when the patient is in the supine position [8,10,11,19]. It has been shown that there is a change in the cardiac axis between prone and supine positioning of approximately 9 degrees in the trans-axial plane [20]. This change correlated with differences in cardiac wall activity between supine and prone position, indicating that the attenuation in the heart itself (myocardium and blood pool) could be a contributing factor. The level of the left hemi-diaphragm and the visceral organs are also altered, which may change the attenuation pattern.

Inferior attenuation artifacts are known to be most common in male patients [3]. This was seen also in this study, in which 22 of the 26 defects among the men were located inferiorly. Among the women, however, one-third of the defects were also located inferiorly. Inferior defects disappeared in prone images to a great extent (71%) in the whole study population, and in all but one of the women. This is in agreement with a recent study by Nishina and colleagues, which showed significantly higher myocardial uptake in inferior segments in the prone versus supine position in both men and women [19].

Attenuation artifacts in the anterior wall were predominantly seen in women. Only 35% of the anterior defects disappeared in the prone position in this study, which is considerably less than the disappearance rate for inferior defects. The left breast, which overlies the left ventricular myocardium, probably contributes to these attenuation artifacts [3,21].

Of the 63 defects on supine imaging, 26 remained on prone imaging. Only 2 of these persistent defects was a defect caused by ischemic myocardial infarction as determined by CMR. This suggests that prone imaging is not always sufficient to differentiate between attenuation artifacts and myocardial infarction.

However, there were 2 patients with remaining defects in the prone position that did not have myocardial infarction in that area confirmed by MRI, but showed signs of thinned myocardium and depressed regional myocardial function or epicardial fibrosis. This would suggest that these patients had a true decrease in myocardial perfusion in that area showed by SPECT. No patients with defects that disappeared in the prone position showed regional dysfunction or epicardial scarring on the MRI.

In patients without previously documented coronary artery disease, it is important to be able to confirm or exclude myocardial infarction, since patients with infarction should enter a more aggressive medical treatment. CMR can distinguish between infarctions/injuries and artifacts and might be used in further evaluation of SPECT defects, as suggested by McCrohon et al [22].

In this study, 3 patients had small areas of fibrosis/infarction by CMR that were not visible by myocardial perfusion SPECT imaging. One of these had a subendocardial location which indicates ischemic origin, and hence could represent silent minor myocardial infarction. The other two were represented by epicardial fibrosis which would rather suggest inflammatory origin e.g. perimyocarditis. Such small myocardial injuries could not be expected to be detected by SPECT, since the spatial resolution is lower than that of CMR [23]. The existence of these areas of myocardial fibrosis/infarction missed by SPECT, however, is an important finding.

In our study population with patients without recorded myocardial infarction and a mean age of 59 years, 15% showed fibrosis/infarction on MRI. These findings are in line with the results from 2 recent MRI studies which showed that unrecognized myocardial scars are more frequent than earlier expected in elderly. In approximately 20% in a 70 year old population with no prior history of infarction, myocardial scar was found by CMR [24,25]. Furthermore the origin of the scars may quite often not be associated with atherosclerosis [24,25].

Various systems, such as those using radioactive substances as external transmission sources or computed tomography (CT), can provide attenuation maps for correction [19]. These systems are improving, but they do not always provide accurate correction, and not all hospitals have access to them [26].

The use of computed tomography (CT) for transmission scanning has proven to be of value for correction of attenuation artifacts in SPECT imaging [27]. CT can also be used concomitantly for calcium scoring sometimes in hybrid settings [28]. However, these systems have disadvantages in the potential for misregistration of emission and transmission scans due to the fact that the images are obtained sequentially [29]. This may happen in the case of patient movement or if the device is improperly positioned.

The advantages of using prone acquisition to detect attenuation artifacts are that no extra hardware or software is needed, it is inexpensive, and it does not deliver any extra radiation to the patient. The risk of technical misuse and software bugs which would affect the result is minimized, as there are no extra devices or software. [30].

Using wall motion and thickening in the ECG gated SPECT acquisition has been proposed as a means for differentiation between a true defect and attenuation. In this study this would have led to a relatively high number of false positive indications of fibrosis/infarction while at the same time some of the true defects would have been missed (Table 2).

There are some limitations to the study; one is the fairly limited number of included patients. However, using MRI as gold standard in this study showed that all disappearing defects exclude infarction or fibrosis in that area. This indicates a correct validation of attenuation defects even with this number of patients

No quantitative measurements such as summed stress score (SSS) nor summed thickening score (STS) were used. However, visual analysis of gated SPECT images is used in the clinical setting and therefore the study was designed accordingly.

It may be argued that severe stenosis with no coronary flow reserve (hibernating myocardium) can explain fixed defects at SPECT. Therefore, cine MR images were analyzed in addition to the delayed enhancement (DE) MRI. Hibernating myocardium will be detected as non-infarcted regions on DE-MRI with decreased regional wall motion on cine MRI and a perfusion defect on SPECT. In our material there were no patients with signs of hibernating myocardium on the combined MRI and SPECT images. Therefore there was no need for coronary angiography data in this study

## Conclusion

Defects observed in this study on supine SPECT imaging that disappeared in the prone position were likely caused by attenuation artifacts and not by myocardial fibrosis/infarction. This finding could help to rule out ischemic heart disease for a number of patients admitted for SPECT examination, preventing unnecessary further investigations and treatment, and also indicating a better prognosis. This would save time, money and enhance the diagnostic accuracy, while at the same time saving patients from the risk involved in unnecessary tests.

## Competing interests

The authors declare that they have no competing interests.

## Authors' contributions

The authors have contributed to the study in the following way: BH conceived and designed the study, collected patients, read and interpreted data and was the main responsible for the writing and revision of the manuscript. EP conceived and designed the study, collected patients, read and interpreted data and participated in the writing of the manuscript. MC conceived and designed the study, read and interpreted data and participated in the writing and revision of the manuscript. OP conceived and designed the study, read and interpreted data and participated in the writing of the manuscript. HA conceived and designed the study, read and interpreted data and participated in the writing and revision of the manuscript. All authors have read and approved the final manuscript.

## Pre-publication history

The pre-publication history for this paper can be accessed here:

http://www.biomedcentral.com/1471-2342/9/16/prepub
